# Neurophysiological markers of asymmetric emotional contagion: implications for organizational contexts

**DOI:** 10.3389/fnint.2024.1321130

**Published:** 2024-01-30

**Authors:** Sarah Boukarras, Donato Ferri, Laura Borgogni, Salvatore Maria Aglioti

**Affiliations:** ^1^Department of Psychology, Sapienza University of Rome, Rome, Italy; ^2^Santa Lucia Foundation, IRCCS, Rome, Italy; ^3^Ernst and Young (EY), Rome, Italy; ^4^Sapienza University of Rome and CLN2S@Sapienza, Italian Institute of Technology, Rome, Italy

**Keywords:** organizational neuroscience techniques, interpersonal neuroscience, hyperscanning, leadership, physiological synchronization, time lag analysis, emotional contagion

## Abstract

Emotions play a vital role within organizations, impacting various crucial aspects of work such as job satisfaction, performance, and employee well-being. Understanding how emotional states spread in organizational settings is therefore essential. Recent studies have highlighted that a leader’s emotional state can influence their followers, with significant consequences on job performance. Leaders thus possess the ability to influence their employees’ psychological state and, consequently, their well-being. However, the biological underpinnings of emotional contagion from leaders to followers remain unexplored. The field of interpersonal (neuro)physiology, which involves recording brain and peripheral activity of multiple individuals during interactions, holds great potential for investigating this phenomenon. Analyzing the time-lagged synchronization of neurophysiological activity during interactions may serve as a measure of the leader’s influence on their followers in organizational contexts. In this “mini review,” we examine empirical studies that have employed interpersonal (neuro)physiology to quantify the asymmetrical contagion of emotions in different contexts. Asymmetrical contagion was operationalized as the unidirectional influence exerted by one individual (i.e., the “sender”) to another one (i.e., the “receiver”), whereby the receiver’s state can be predicted by the sender’s one. The reviewed literature reveals that delayed synchronization of physiological states is a widespread phenomenon that may underpin the transmission of emotions. These findings have significant implications for various aspects of organizational life, including leader-to-employee communication, and could drive the development of effective leadership training programs. We propose that Organizational Neuroscience may benefit from including interpersonal neurophysiology in its methodological toolkit for laboratory and field studies of leader-follower dynamics.

## Introduction

### Emotions in organizations

Emotions impact working life at the individual, group and organization level (Ashkanasy, 2003) and can produce important consequences on employees’ productivity and well-being (Barsade et al., 2018). Emotions and moods, far from being exclusively private experiences, are shared and spread among individuals during social interactions, giving rise to “collective emotions” (Barsade et al., 2018; Goldenberg et al., 2020). The mechanisms underlying the spread of emotions and moods in organizations have received increasing attention in recent years, with a particular emphasis on the leader-to-follower dynamics. The expression of positive emotions by a leader can, in fact, influence the followers’ mood (Sy et al., 2005; Bono and Ilies, 2006; Sy and Choi, 2013) and can benefit the group’s performance (Barsade, 2002; Visser et al., 2013). In addition, positive emotional contagion is one of the defining features of charismatic (Antonakis et al., 2016) and transformational (Bass and Riggio, 2006) leadership and a leader’s positive mood can increase the follower’s perception of his or her charisma (Cherulnik et al., 2001; Erez et al., 2008; Cheng et al., 2012). Conversely, the transmission of negative emotional states from a leader can disrupt group coordination (Sy et al., 2005).

### Physiological synchrony as a window on emotional contagion

An accredited neuroscientific model for explaining the mechanisms of emotional contagion (Niedenthal and Brauer, 2012) implies simulative processes through which one experiences the same emotion observed in another person, ultimately converging with them at the phenomenological and physiological level (Hatfield et al., 1993; Hess and Blairy, 2001; Prochazkova and Kret, 2017). Recent methodological and theoretical advances in social neuroscience have opened the way for measuring this convergence (i.e., neurophysiological synchrony) via the simultaneous recording of brain and autonomic activity in two or more individuals (Babiloni et al., 2006; Dumas et al., 2010; Palumbo et al., 2017; Czeszumski et al., 2020; Mayo et al., 2021). When dyad or group members can be distinguished according to intrinsic or assigned characteristics (i.e., gender, role, status, see Kenny et al., 2006), physiological or neural synchrony may follow specific temporal patterns that are compatible with directional influence (i.e., from person A to person B or *vice-versa*). Physiological “contagion” or “influence” (Thorson and West, 2018) refers to the phenomenon wherein the autonomic or brain activity of one individual (i.e., the “sender”) at time tX predicts the activity of another individual (i.e., the “receiver”) at a later time tX + t. This mini review article scrutinizes the available literature on neurophysiological contagion in different asymmetric relationships, with a special focus on the transmission of emotional states. To this end, we selected experimental studies in which a delayed synchronization of brain or peripheral activity was measured in dyads or groups including parent–child dyads, romantic couples, psychotherapist- and clinician-patient dyads, unacquainted dyads, and groups.

### Measures of neurophysiological contagion

A wide range of analytical methods exist and have been used for quantifying neurophysiological contagion. While a detailed description goes beyond the scope of this article (the interested reader is referred to specialized reviews, e.g., Helm et al., 2018; Thorson et al., 2018; Czeszumski et al., 2020; Dumas and Fairhurst, 2021), here we provide a summary of the methods used in the reviewed articles (see also Table 1). A broad distinction can be made between linear models and time series analyses (also defined as “nomothetic” and “idiographic” methods, see Palumbo et al., 2017), the former referring to variations of regression models applied at the group level and the latter to methods for assessing the relationship between individual (i.e., at the dyad level) time series. In linear models, neurophysiological data is generally averaged over the entire recording block or in smaller time bins, then the (mean) activity of one individual is regressed with the partner’s activity (see Table 1) at a subsequent time lag. These models often include as predictors also the experimental condition(s) (e.g., the type of interaction or manipulation). Time series models are instead usually computed over the entire recording block to derive a single value of correlation, causality or coherence for each dyad or block. Directional synchrony is computed by shifting the series of a predetermined time lag (i.e., cross-correlation). It is important to note that neurophysiological time series are usually characterized by autocorrelation (i.e., the correlation between present and past data points from the same series at determined time lags), which can produce spurious results when computing cross-correlation (see Dean and Dunsmuir, 2016; Thorson et al., 2018). For this reason, researchers interested in measuring neurophysiological contagion should always ensure that their models control for stability and autocorrelation in their individual series (Helm et al., 2018; Thorson et al., 2018).

**Table 1:** List of statistical methods applied for quantifying asymmetric contagion in the reviewed studies.

Family of method	Description	Examples
Linear models	Statistical models in which the physiological activity of one of the participants is included as the dependent variable, while the other participant is considered as a regressor or predictor. In some cases, the authors have included a time lag or regressed the predictor at a subsequent time lag with the dependent variable at time zero.	Growth curve model
		Hierarchical linear Model
		Multilevel cross-classified model
		Multilevel structural equation model.
		Two-level crossed model
		Stability and influence model
Time series analysis	Models assessing the relationship between two time series (correlation or causality) above and beyond each series autocorrelation. PDC and WTC are applied to the frequency domain.	Cross-correlation
		Granger causality (GC)
		Partial directed coherence (PDC)
		Wavelet transform coherence (WTC)
		Cross-brain connectivity (CBC)

The article is divided in two sections, dedicated to physiological (i.e., autonomic) and neural contagion. Table 2 offers a summary of the autonomic and neural measures collected in the reviewed papers.

**Table 2:** Summary of the neurophysiological measures reported in the reviewed articles.

	Technique	Description and functional significance of recorded measures
Autonomic nervous system	Electrocardiography (ECG)	Heart rate (HR) - Number of heart beats per minute. Increased HR is considered as a marker of sympathetic activation and/or parasympathetic deactivation.
		Pre-ejection period (PEP) -Time between the Q wave and the opening of the aortic valve (measured with impedance cardiography). PEP is a pure measure of sympathetic activation.
		Respiratory sinus arrythmia (RSA) - Respiration-driven increase and decrease (variability) in HR. RSA is a reliable index of parasympathetic activity.
	Electrodermal activity (EDA)	Skin conductance (SC) - Changes in the electrical activation of sweat glands measured from the skin. SC is a pure measure of sympathetic activation.
	functional Infrared Thermal Imaging (fITI)	Nose tip temperature - temperature decrease is related to sympathetic alpha-adrenergic vasoconstrictor activity
Brain	Electroencephalography (EEG)	Oscillatory electrical activity of neurons. Power and frequency of the oscillations reveal the activation of brain areas.
	functional magnetic resonance (fMRI)	Brain blood-oxygen-level-dependent (BOLD) activity as a measure of brain areas activation.
	functional near-infrared spectroscopy (fNIRS)	Measures BOLD activity using a portable device

## Physiological contagion (autonomic nervous system)

### Parent–child interactions

Early interactions with the caregiver are thought to provide a scaffolding for the development of the self (Bolis and Schilbach, 2020; Fini et al., 2023), and bio-behavioral synchrony seems to play a special role in this process (Feldman, 2007; Bolis and Schilbach, 2017). Studies have shown that mother and child physiological activity becomes synchronized during face-to-face interaction (Feldman et al., 2011) and that this (concurrent) synchrony can reveal the dynamics of mother-to-child stress contagion (Waters et al., 2014). Yet only few studies have explicitly examined directional physiological influence. In one of these, authoritative parents’ RSA (see Table 2) was found to predict the children’s during discussions on conflicting topics (Oshri et al., 2021). The effect was further intensified by the children’s level of sympathetic arousal, hinting at a non-passive role of the receiver (i.e., the child) in the contagion process. In other cases, the child itself can play the role of sender, as was observed in a study measuring lagged synchrony between the nose tip temperature (reflecting the activation of the sympathetic nervous system, see Table 2) of children and their mothers (Manini et al., 2013). The transmission of physiological states can happen also in non-emotional contexts. Armstrong-Carter and collaborators (Armstrong-Carter et al., 2021) tested parents and children during different dyadic interactions and observed that the parent’s RSA predicted that of the children during a problem-solving task. Adverse events, such as adoption or foster care, may instead impair the parents’ ability to attune with their children, leading to a reduction in child-to-parent physiological contagion (Callaghan et al., 2021).

### Romantic couples

Emotional convergence plays a fundamental role in strengthening romantic relationships by increasing mutual alignment in thoughts and feelings (Anderson et al., 2003; Mazzuca et al., 2019). The seminal study of Levenson and Gottman (Levenson and Gottman, 1983) was the first to observe the synchronization of HR and SCR activity in husbands and wives during couple therapy. Measuring physiological contagion in romantic couples can be informative of which member is driving the interaction and who has more influence over the other (i.e., the male or the female member). Depending on the context, both men and women seem to be able to influence each other’s physiology. Ferrer and Helm (2013) observed that, during an imitation task, changes in the male partner’s HR followed those of the female partner, while the opposite pattern was observed in a study measuring HR and SCR lagged synchrony in married couples during a conflict-solving interaction (Thomsen and Gilbert, 1998). In another case, lagged synchronization was observed between both men and women’s RSA and their partners’ previous RSA, with higher synchronization in couples exhibiting better relationship satisfaction (Helm et al., 2014).

### Psychotherapy and clinician-patient interaction

Interactions between clinicians (e.g., psychotherapist, medical doctor) and patients are quintessentially asymmetric due to differences in knowledge and power distribution and therefore are particularly suited for the investigation of physiological contagion. Nevertheless, there is evidence that both the patient and the clinician can be identified as “senders.” Psychotherapist-to-patient physiological contagion (but not the reversed pattern) was found during mock psychotherapy sessions, and it was higher in dyads including therapists with a secure attachment style (Palmieri et al., 2018). Similarly, oncologist-to-patient contagion was observed during consultations for cancer treatment (Vigier et al., 2021). Conversely, patient-to-clinician contagion was observed in psychotherapy, but only with trained therapists (Messina et al., 2013). In an interesting attempt to move beyond dyadic relations, Thorson et al. (2021c) conducted a study in therapy groups for methamphetamine use disorder, where groups received oxytocin or placebo before therapy sessions. In the oxytocin groups, cardiac reactivity of participants could be predicted by their mates’ activity, indicating physiological contagion between facilitators and patients, although the linkage flowed in both directions.

### Unacquainted dyads and groups, ethnicity and social status effects

Asymmetric interactions can occur between unacquainted individuals, for example when they are assigned different roles by the experimenters or when they are sampled from different ethnic groups. The latter case is exemplified by the study of West and collaborators (West et al., 2017), where (sympathetic) physiological contagion from European Americans to African Americans was observed in dyads where the European American partner showed higher anxiety for inter-racial encounters, suggesting the influence of negative expectations on physiological linkage. Stress contagion can occur not only between mothers and children but also between strangers. Empathy seems to play an important role in this process, as indicated by the results of a study (Brown et al., 2021) measuring physiological contagion in dyads where one member (the “experiencer”) was assigned to undergo a stressful task and to disclose negative personal experiences to their partner (the “listener”). One intriguing question is whether physical presence is strictly necessary for stress contagion to occur. There is evidence that this is not the case, as Dimitroff et al. (2017) observed a lagged synchronization between the HR of participants observing videos of strangers undergoing a stressful procedure and the HR of the strangers themselves. Social status, or power, can play a role in determining the direction of physiological contagion, as shown in Kraus and Mendes (2014) study. The experimenters manipulated the social status of participants leading a mock negotiation by making them wear either upper- or lower-class clothes (i.e., a suit or sport clothes). When participants wore high-status clothes, their PEP values predicted that of the other participant with a 30 s lag, indicating that high-status individuals may catalyze the sympathetic activity of people they are interacting with. However, another experiment manipulating social status revealed that also the opposite might be true (Thorson et al., 2021b). In this study, one group member was assigned higher status than the others, and results showed that the RSA of high-status women who were successful in influencing the group choice could be predicted by the RSA of their female groupmates, suggesting that effective leadership may come through the leaders’ ability to get attuned with their followers’ psycho-physiological state. It should be noted, however, that this finding could not be replicated in a later, similar, study (Thorson et al., 2021a).

## Neural contagion (central nervous system)

### Parent–child interactions

Hyperscanning techniques are increasingly used for investigating the dynamics of parent–child interactions, especially since the adoption of portable brain imaging technologies such as fNIRS (Nguyen et al., 2021a,b). However, studies explicitly designed to measure directional inter-brain synchrony are limited, and most of them did not investigate the transmission of emotional states. In one of them, using fMRI (see Table 2), Ratliff et al. (2021) measured time-lagged cross-brain connectivity (CBC, see Table 1) between the BOLD signal time series of adolescents and parents discussing conflicting topics, and found a parent-driven connectivity in multiple emotion-related brain regions, in line with the results from the Oshri et al. (2021) study. In addition, the amount of CBC between parent cortical regions and adolescent bilateral anterior insula was correlated with reduced supportive behavior in the parent. In non-emotional contexts, mother-led synchronization of the temporo-parietal junction (TPJ, a brain area involved in mentalizing and perspective taking) was observed during a free play task using fNIRS (Zhao et al., 2021). Moreover, TPJ synchronization was associated with the child’s responsiveness to the interaction behaviors of the adult, highlighting again (see Oshri et al., 2021) the non-zero impact of the “receiver” on neural contagion. As in the case of physiological contagion (Dimitroff et al., 2017), also neural contagion can occur without a physical interaction taking place. Leong and colleagues (Leong et al., 2017) applied dual EEG to parent–child dyads and found that alpha and theta frequency activity of adults singing a song granger caused (see Table 1) the alpha and theta activity of children observing their parents either live or in a video recording. In both cases, the synchronization was stronger if the adult gaze was directed toward the children.

### Romantic couples

Non-directional inter-brain synchrony in romantic couples has been found to be significantly higher than in control dyads across different tasks and neural measures (Kinreich et al., 2017; Djalovski et al., 2021). Hypotheses regarding directionality have, however, been tested in few studies. In one of them, Long and colleagues (Long et al., 2022) measured the brain activity of romantic partners during a hand-holding task using dual fNIRS and found a woman-led synchronization. Similar results were obtained in another fNIRS study, which tested romantic couples during a cooperation task (Pan et al., 2017), revealing a stronger directional coupling in the superior frontal cortices from females to males than vice versa. Conversely, Zhang and colleagues (Zhang et al., 2023) found stronger male-driven inter-brain synchronization during interpersonal emotion regulation when males used cognitive engagement as a strategy. Overall, as in the case of physiological contagion (Thomsen and Gilbert, 1998; Ferrer and Helm, 2013; Helm et al., 2014), data from studies using neural measures indicate the presence of delayed synchronization of brain states between romantic partners, which can flow from either the male to the female or vice versa.

### Psychotherapy and clinician-patient interactions

Lagged inter-brain synchrony also occurs during therapist-patient interactions. One study applied dual fNIRS in client-counselor dyads involved in counseling sessions (Zhang et al., 2020) and found that activity in the counselor rTPJ predicted the clients’ activity in the same area. In addition, the synchronysation was higher when the counselor was an expert compared to when he or she was a novice, highlighting again, as in (Messina et al., 2013), the role of clinician’s expertise in the emergence of neural contagion. Finally, Ellingsen and colleagues (Ellingsen et al., 2020) investigated patient-clinician dyads during a pain treatment session using fMRI. They observed a delayed, patient-led, concordance between the activity of the rTPJ in patients and clinicians, associated with stronger therapeutic alliance and analgesia following treatment. A second analysis examined the relationship between neural concordance and reciprocal dynamics of facial expressions (Ellingsen et al., 2022) and found that clinician’s ability to mirror patients’ painful expressions predicted neural concordance and therapeutic alliance. These results fit with the ones observed by Messina and colleagues (Messina et al., 2013) highlighting the role of the clinician’s empathic abilities in determining neurophysiological contagion.

### Unacquainted dyads, leader emergence, education, and emotional contagion

Two of the first hyperscanning experiments ever conducted involved recording EEG from groups of people engaged in a card game and examining the direction of flow in brain activity using granger causality. Relevant for the present work, is the finding that oscillatory activity in the brain of the first player (i.e., the one who drew the first card) “granger caused” (see Table 1) the activity of the other players’ brain (Babiloni et al., 2006; Astolfi et al., 2010). Similar results from the same research group were observed in professional flight pilots, with EEG activity of the first officer “granger causing” that of the captain (Astolfi et al., 2011). Using fNIRS, in their seminal study, Jiang and colleagues (Jiang et al., 2015) recorded brain activity in groups of three participants who were engaged in a group discussion. During the experiment, one of the three participants spontaneously emerged as a leader, i.e., started to guide the conversation. The authors observed a significantly higher neural synchronization between leaders and followers than between followers and followers, and Granger Causality (see Table 1) analysis showed that mean causality was higher from the leaders to the followers than vice versa. Similarly, Pan and collaborators (Pan et al., 2018) measured brain activity with fNIRS from learner-instructor dyads and observed that the activity of the inferior frontal cortex of the instructor “granger caused” that of the learner.

## Discussion

The diverse research discussed in this mini review highlights the presence of neurophysiological contagion during asymmetric social interactions. Asymmetric roles may be structural, as in mother–child and clinician-patient interactions, or situational, as in the case of who moves first in the game (Babiloni et al., 2006; Astolfi et al., 2010), or who is assigned/receives the higher status (Jiang et al., 2015; Thorson et al., 2021b).

Neurophysiological contagion is, in some cases, associated with the spread of emotional states, in particular negative ones like stress (Manini et al., 2013; Waters et al., 2014; Ellingsen et al., 2020; Oshri et al., 2021; Ratliff et al., 2021). Stress is a major issue in organizational settings, not only impairing work-related performance but also contributing to various health issues like heart disease and high blood pressure (Siu et al., 2007), and even increasing cancer risk (Yang et al., 2019). Interpersonal neurophysiology might thus become a powerful tool for shedding light on the dynamics of stress contagion in the workplace. Neurophysiological contagion also occurs during positive interactions, like affective touch (Long et al., 2022) and thus might be associated with the spread of positive states, although positive contagion is generally less studied (see Morelli et al., 2015; Mello et al., 2023).

It is important to notice that neurophysiological contagion does not only occur in emotional contexts but can also be associated with neutral interactions (Thomsen and Gilbert, 1998; Babiloni et al., 2006; Astolfi et al., 2010; Armstrong-Carter et al., 2021). Thus, interpersonal neurophysiology might be applied to the investigation of learning processes and cooperation in the workplace. Relevant for the present work, neurophysiological contagion occurs in leader-follower (Jiang et al., 2015) and status-based (Kraus and Mendes, 2014; Thorson et al., 2021b) dynamics. It is plausible that the increased attention received by higher status individuals (Shepherd et al., 2006; Dalmaso et al., 2012) may be the mechanism explaining asymmetric contagion. The possibility of exploiting interpersonal neurophysiology for tracking the emergence of a leader in unstructured groups or for assessing the effectiveness of leadership strategies in organizations is plausible. Since leaders seem to transmit their emotional states to their followers (Barsade, 2002; Sy et al., 2005; Sy and Choi, 2013), it is likely that also neurophysiological contagion would be observed in leader-follower interactions. In this sense, interpersonal neurophysiology might be leveraged for investigating how employees are influenced by their leader’s mood and emotions. However, although one could expect that neurophysiological contagion tends to flow from the individual who has more power (i.e., parent, clinician, teacher, high-status) to the less powerful, this is not always the case. In fact, there seem to be instances where neurophysiological contagion is started by children, patients or lower-status individuals (Messina et al., 2013; Callaghan et al., 2021; Thorson et al., 2021b). Thus, it might be the case that the “sender” is better identified as the person who is expressing the strongest emotion, as in the case of nervous Caucasians interacting with Black people (West et al., 2017), distressed children (Waters et al., 2014) or adults (Dimitroff et al., 2017; Brown et al., 2021) and suffering patients (Messina et al., 2013; Ellingsen et al., 2020), rather than the person who has more power. The *a priori* assumption that the leader will always be identified as the “sender” could induce researchers to miss important information regarding the role played by the followers. To avoid this potential problem, researchers should always measure physiological contagion in both directions. Moreover, even when neurophysiological contagion flows in the expected direction (i.e., from the adult to the child, from high- to low-status individuals), there is evidence that the receiver plays an active role in the process. Indeed, levels of arousal (Oshri et al., 2021), expertise (Messina et al., 2013), empathy (Brown et al., 2021; Ellingsen et al., 2022) and responsiveness (Zhao et al., 2021) measured in the receiver do modulate the magnitude of lagged synchrony and should always be controlled for.

Another aspect deserving discussion pertains to the content that is transmitted through contagion. Autonomic and neural markers can be used to infer the presence and intensity of emotional arousal, but identifying the specific emotion that the participant is experiencing from his physiology might be challenging (Lench et al., 2011; Lindquist et al., 2012). Behavioral and subjective data complementing the neurophysiological are needed to understand what kind of emotional states are transmitted from one individual to another. Interestingly, some of the reviewed studies (Dimitroff et al., 2017; Leong et al., 2017) reported the presence of neurophysiological contagion in video-mediated interactions, suggesting that individuals can achieve neurophysiological alignment with others, despite physical separation and temporal differences, through the overt display of social and emotional signals. This has important implications for organizational research and practice, considering that in recent years, there has been a remarkable increase in the integration of video communication tools in organizational settings, paralleled by a widespread transition to remote work among professionals.

In conclusion, we suggest that the measurement of asymmetric physiological contagion might be incorporated into the rising field of organizational neuroscience to shed light on the interpersonal dynamics (see Boukarras et al., 2022) occurring in the workplace.

## Author contributions

SB: Conceptualization, Investigation, Writing – original draft. DF: Writing – review & editing. LB: Supervision, Writing – review & editing. SA: Conceptualization, Resources, Supervision, Writing – review & editing.
